# A barrier to homologous recombination between sympatric strains of the cooperative soil bacterium *Myxococcus xanthus*

**DOI:** 10.1038/ismej.2016.34

**Published:** 2016-04-05

**Authors:** Sébastien Wielgoss, Xavier Didelot, Roy R Chaudhuri, Xuan Liu, Gareth D Weedall, Gregory J Velicer, Michiel Vos

**Affiliations:** 1 Department of Environmental Systems Science, Institute of Integrative Biology, ETH Zürich, Zürich, Switzerland; 2 Department of Infectious Disease Epidemiology, Imperial College London, London, UK; 3 Centre for Genomic Research, University of Liverpool, Liverpool, UK; 4 Department of Molecular Biology and Biotechnology, University of Sheffield, Sheffield, UK; 5 Department of Vector Biology, Liverpool School of Tropical Medicine, Liverpool, UK; 6 European Centre for Environmental and Human Health, Medical School, University of Exeter, Penryn, UK

## Abstract

The bacterium *Myxococcus xanthus* glides through soil in search of prey microbes, but when food sources run out, cells cooperatively construct and sporulate within multicellular fruiting bodies. *M. xanthus s*trains isolated from a 16 × 16-cm-scale patch of soil were previously shown to have diversified into many distinct compatibility types that are distinguished by the failure of swarming colonies to merge upon encounter. We sequenced the genomes of 22 isolates from this population belonging to the two most frequently occurring multilocus sequence type (MLST) clades to trace patterns of incipient genomic divergence, specifically related to social divergence. Although homologous recombination occurs frequently within the two MLST clades, we find an almost complete absence of recombination events between them. As the two clades are very closely related and live in sympatry, either ecological or genetic barriers must reduce genetic exchange between them. We find that the rate of change in the accessory genome is greater than the rate of amino-acid substitution in the core genome. We identify a large genomic tract that consistently differs between isolates that do not freely merge and therefore is a candidate region for harbouring gene(s) responsible for self/non-self discrimination.

## Introduction

*Myxococcus xanthus* is the best-studied member of the order Myxococcales (delta-proteobacteria), members of which are well known for their ability to construct multicellular fruiting bodies (Whitworth, 2008). *M. xanthus* occurs in a wide range of soil habitats, where it uses gliding motility to swarm in groups in search of organic compounds, including other bacteria, to digest using a cocktail of extracellular enzymes (Berleman and Kirby, 2009). When nutrients become scarce, social groups undergo multicellular development to form fruiting bodies, within which a minority of cells form stress-resistant spores, whereas the majority of cells either lyse (Shimkets, 1990) or remain as vegetative cells at the fruiting body periphery.

Although the genetic bases of *M. xanthus* behaviours are being steadily unravelled, primarily through the study of two lab strains, DK1622 (Goldman *et al.*, 2006) and DZ2 (Müller *et al.*, 2013), gaining insights into the evolution of bacterial sociality and multicellularity crucially depends on the study of natural variation within species. In a previous study, 78 *M. xanthus* clones were isolated from a 16 × 16-cm grid in Tübingen, Germany (Vos and Velicer, 2006). Sequencing of multiple housekeeping loci revealed a total of 21 multilocus sequence type genotypes and subsequent phenotypic assays revealed significant diversity in a wide range of phenotypes, including motility (Vos and Velicer, 2008b), secondary metabolite production (Krug *et al.*, 2008), predation (Morgan *et al.*, 2010), social development (Kraemer *et al.*, 2010) and social interaction phenotypes (Vos and Velicer, 2009).

This study focuses on whole-genome data from 22 *M. xanthus* isolates belonging to the two most common multilocus sequence type clades from the Tübingen centimetre-scale population, which we will refer to as clade I and clade V, corresponding to the original publication describing these strains (Vos and Velicer, 2006). On the basis of five housekeeping gene fragments sequenced in one representative of each clade (Vos and Velicer, 2008a), the two clades are ~99.7% identical at the nucleotide level. No nucleotide differences were found between isolates within each clade based on three housekeeping gene fragments (Vos and Velicer, 2006). However, it was clear that not all isolates within each lineage are completely genetically identical because when swarming on agar, some isolates belonging to the same genotype form boundaries between each other in a heritable and reproducible manner (Vos and Velicer, 2009). The high diversity of this self/non-self-discrimination system has been hypothesised to have a role in protecting clonal swarms of cooperative genotypes from invasion by freeloading genotypes from neighbouring social groups (Velicer and Vos, 2009; Vos and Velicer, 2009). The genetic basis of this interaction phenotype is not known, but is phenotypically similar to swarm boundaries characterized in the gamma-proteobacterium *Proteus mirabilis* (Gibbs *et al.*, 2008; Alteri *et al.*, 2013) and *Bacillus subtilis* (Stefanic *et al.*, 2015).

Relatively simple lab systems using experimentally evolved or genetically engineered clones have been able to provide profound new insights into specific social behaviours of microorganisms as well as the evolution of sociality in general (West *et al.*, 2006). However, to understand how social traits evolve, it is vital to study social diversity in natural populations of coexisting clones. In particular, comparison of closely related isolates that live in sympatry allows reliable inference regarding the fine-scale ‘ingredients' of (social) evolutionary processes: mutation, homologous recombination, changes in gene content, genetic drift and natural selection. Moreover, this approach ensures that there are no confounding effects of isolation methodology, isolation time, biogeography or (macroscale) habitat differences (Vos and Velicer, 2008b).

Here we investigate patterns of genomic divergence between and within two very closely related clades of *M. xanthus* isolates inhabiting the same centimetre-scale soil habitat. We test whether barriers to recombination (gene flow) exist between both clades, which might facilitate ecological divergence and coexistence, and, at longer time scales, potentially even speciation (Vos, 2011; Cadillo-Quiroz *et al.*, 2012; Shapiro *et al.*, 2012; Shapiro and Polz, 2014). We next examine whether divergence primarily comes in the form of substitutions in the core genome or changes in the accessory genome (due to lateral gene transfer, gene duplication and gene loss). We identify substantial variation in the accessory genome of this population, and in particular focus on a ~150-kB region in which gene-content variation matches swarming incompatibilities (Vos and Velicer, 2009).

## Materials and methods

### Strains, DNA isolation and sequencing

This study focuses on 22 *M. xanthus* isolates (clade I: A00, A06, A07, A26, A32, A39, A46, A49, A58, A60, A64 and A92, and clade V: A15, A30, A31, A34, A44, A51, A56, A62, A72 and A93) isolated in 2003 from a centimetre-scale population in Tübingen, Germany (48° 32′ N, 9° 3′ E), as has been previously described (Vos and Velicer, 2006). DNA was isolated from cultured cells using Qiagen's Genome Extraction (Qiagen, Hilden, Germany) protocol for bacteria and Genomic Tips G/100 according to the manufacturers recommendations. Illumina HiSeq 2000 sequencing (Illumina, San Diego, CA, USA; 2 × 100 bp paired-end reads with an average insert size of 500 bp) was performed by the Beijing Genomics Institute (BGI). Adaptor sequences, read duplicates and low-quality reads were filtered, yielding a total of >10 000 000 high-quality reads per genome (a net coverage of ~100 sequences per base pair; Supplementary Table 1). Alignments of reads to the reference genome of *M. xanthus* DK 1622 (GenBank accession CP000113) were visualised using Integrative Genomics Viewer (Thorvaldsdóttir *et al.*, 2012). Sequence data are made available in the NCBI Short Read Archive under Accession Number SRP071343.

### Phylogenetics and population genomics analyses

For the whole-genome-based phylogeny, the filtered, paired reads were merged and aligned against all the outgroup, DK1622 (overall tree topology, based on ~6.9 Mbp), the *de novo* assembled clone A00 (within-clade I phylogeny, based on ~7.6 Mbp) or the *de novo* assembled clone A15 (within-clade V phylogeny, based on ~7.6 Mbp) using the tool REALPHY v1.06 (Bertels *et al.*, 2014). From this alignment, maximum likelihood phylogenies were inferred for all 22 genomes as well as clade I and V separately. The best tree was inferred assuming the General Time Reversal (GTR) model of sequence evolution with a gamma-distribution model of rate heterogeneity, using the parameters ‘raxmlHPC-SSE3 -f -m GTRGAMMA -p 123' in RAxML v8 (Stamatakis, 2014). A total of 1000 pseudo-replicates were generated by applying a bootstrapping approach in RAxML v8 with options ‘raxmlHPC-PTHREADS-SSE3 -T 16 -m GTRGAMMA -p 123 -b 123 -# 1000 –k'. For the population genomics analyses, orthologues were inferred using OrthoMCL (Li *et al.*, 2003), relying on reciprocal best BLAST hits (‘blastp -outfmt 6') implemented in the webserver ODoSE (Vos *et al.*, 2013). Single-copy orthologues were retained and aligned at the protein level using Muscle (Edgar, 2004) implemented in TranslatorX (Abascal *et al.*, 2010). Raw data including individual gene sequences, core genome concatemers, a gene distribution table and a table detailing polymorphism for each individual core genome can be downloaded from http://www.odose.nl/u/michiel/h/22-myxo-genomes-w-annotation. A core genome alignment was constructed using progressiveMauve version 2.3.1 (Darling *et al.*, 2010) with default parameters, and used as input for ChromoPainter and FineStructure (Lawson *et al.*, 2012). Unlike traditional phylogenetic techniques, the FineStructure approach attempts to subdivide a sample into a number of ‘populations' that are unknown *a priori*. First, a co-ancestry matrix is built that represents how frequently each individual is most similar to other individuals along the genome. In a next step, individuals with similar co-ancestry profiles are grouped together into populations. The co-ancestry matrix is built assuming that each genome is a mosaic stemming from transfers of gene fragments from different donors. Furthermore, by treating the presence (single or multicopy) and absence of orthologous genes contained in an ~150-kb hotspot region for genomic rearrangements across all 22 A clones as discrete characters, we inferred a maximum parsimony phylogeny using the ‘pars' module in PHYLIP v3.67 (Felsenstein, 2009) with 100 bootstrap replicates. The ACLAME profinder database (http://aclame.ulb.ac.be/Tools/Prophinder/) was used for prophage detection (Leplae *et al.*, 2009).

## Results

### Coexistence of two very closely related *M. xanthus* clades

**Figure 1:**
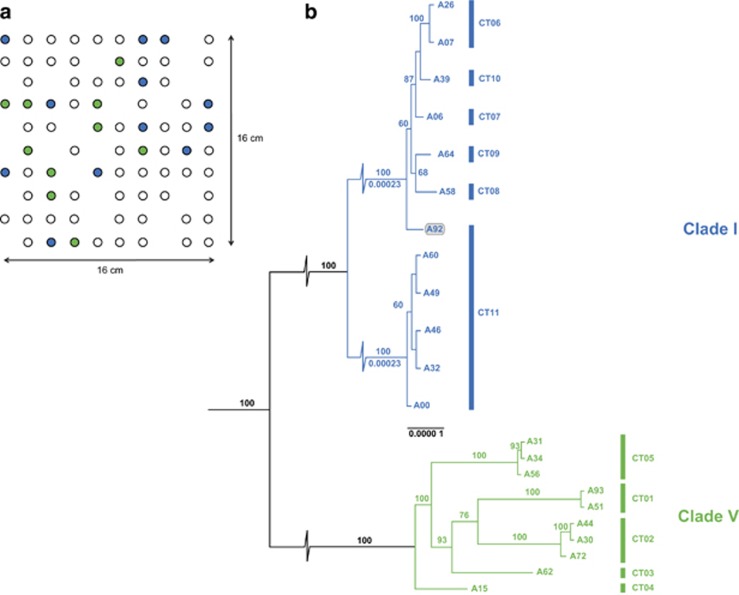
Spatial and phylogenetic relationships of the 22 *M. xanthus* clones. (**a**) Spatial sampling context of the 22 focal A strains. Of 100 samples taken from a 10 × 10 (16 × 16 cm) sampling grid, single *M. xanthus* clones could be isolated from 78 samples (circles). Strains were numbered from left to right, starting with A00 at the top left corner, ending with A99 at the bottom right. Clade I genotypes (blue) and clade V genotypes (green) could initially be distinguished based on the sequencing of three housekeeping genes that were identical within each clade. Both genotypes were the two most common types in the population (15 isolates each). (**b**) Maximum likelihood phylogeny of 22 focal A strains. Whole genomes of 12 members of clade I and 10 clones from clade V were analysed by mapping reads to both the reference strain DK1622 to confirm the overall branching order (black branches), and separately to either A00 (clade I, blue branches) or A15 (clade V, green branches) to increase resolution within clades. The latter subtrees were based on separate phylogenetic analyses and the alignments of 7.6 Mbp of conserved sequence each. Bootstrap values >60% are indicated on top of branches. The very long internal branches in clade I were broken to allow for better visualisation of the short final branches. Branch lengths are indicated below bootstrap values and branch length scale is indicated in substitutions per site. Compatibility-type (CT) grouping for each clone is indicated on the right-hand side. Strain A92 is shown in grey to highlight the incongruence of its phylogenetic position and CT grouping (see text).

The genomes of 22 representatives of two closely related *M. xanthus* clades occurring interspersed in a 16 × 16-cm soil population (Figure 1a; Vos and Velicer, 2006) were sequenced using Illumina HiSeq technology (assembly and annotation statistics are summarised in Supplementary Tables 1–3 and Supplementary Figure 1). The 22 isolates were previously typed on the basis of sequencing multiple housekeeping gene fragments, resulting in the identification of two closely related but distinct clades, designated clades I and V (Vos and Velicer, 2006). We inferred the maximum likelihood tree from the strains' core genome alignment, and confirmed the dichotomy between clades with high confidence. The average degree of sequence dissimilarity between the two clades was ~0.76% per site (52 784 single-nucleotide variants in 6.9 Mb of aligned sequence; Figure 1b). Focusing on each clade separately, clade I consists of two deeply diverged subclades (0.005% substitutions per site), whereas strains in clade V are 10-fold less diverse (0.0005% substitutions per site). Some isolates are extremely closely related; for instance, A31 and A34 do not have a single difference across their aligned sequence of 7.6 Mbp.

The 22 isolates examined here were previously shown to fall into 11 distinct ‘compatibility types' (CTs), defined by the formation of a distinct zone of colony demarcation between two swarms that were initiated from two slightly overlapping liquid cultures pipetted on an agar surface (Vos and Velicer, 2009). Strains belonging to the same CT are phylogenetically closely related (indicated in Figure 1b). The exception is strain A92, which is quite diverged from the other five strains it is compatible with (CT 11).

### Evidence for a barrier to homologous recombination between two closely related sympatric clades

**Figure 2:**
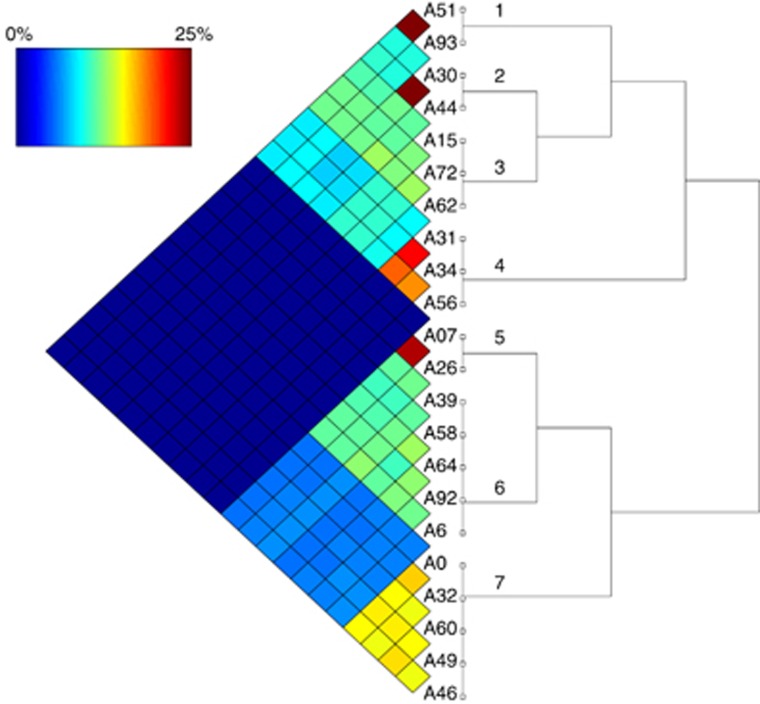
FineStructure analysis of homologous recombination. A co-ancestry matrix is shown on the left, with warmer colours representing higher percentages of recombinational copying from one genome to another. Genomes belonging to the same FineStructure population are connected by a vertical branch in the tree on the right, with the rest of the tree indicating inferred relationships between the seven identified populations.

Homologous recombination rate has been shown to be relatively high in *Myxococcus xanthus* (Vos and Didelot, 2009). To explicitly assess the degree of homologous recombination between and within clades I and V, a FineStructure analysis (Lawson *et al.*, 2012) was applied to an alignment of all core genomes. On the basis of the resulting co-ancestry matrix, FineStructure identified a total of seven ‘populations' defined by inferred ancestry patterns, three in clade I and four in clade V (Figure 2). Population membership was inferred with high confidence (probability>0.99) for all strains (Lawson *et al.*, 2012). The co-ancestry proportion between members of the same population was measured at between 12 and 25% of the genome. Members of different populations within each clade had co-ancestry proportions between 5 and 12% of the genome. In stark contrast, transfer events between members of the two separate clades I and V were negligible (<0.01%), indicating that a strong barrier to homologous recombination evolved between these clades since divergence from their last recent common ancestor.

An additional line of evidence indicates reduced recombination between clades I and V. Homoplasic single-nucleotide polymorphisms (SNPs) present in more than one clade are likely to reflect inter-clade recombination (Maynard Smith and Smith, 1998; Ansari and Didelot, 2014; Everitt *et al.*, 2014), although such patterns may sometimes represent parallel evolution. Interestingly, only 8 out of 83 780 total SNPs in our data set were found to be homoplasies shared by members of both the clades, with the vast majority being present within only one clade. In contrast, nearly twofold as many homoplasic SNPs (14) were present within each of the two deep subclades of clade I (Figure 1b). Because clades I and V are ~10-fold more divergent from one another than are the two clade I subclades, a proportionally greater number of homoplasic SNPs should be present in both clades I and V than in both of the clade I subclades under the null hypothesis that homologous recombination rates are equal across versus within clades I and V. The strong barrier to recombination between clades I and V is thus confirmed based on the number of SNP homoplasies found between the clade I subclades.

### The rate of accessory genome change in *M. xanthus* is higher than the rate of amino-acid substitution in the core genome

**Figure 3:**
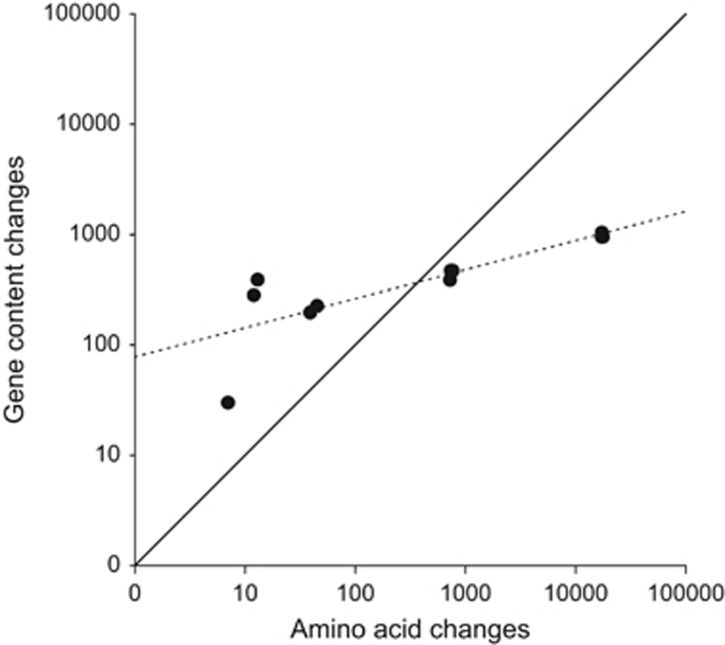
Rate of amino-acid substitution in the core genome versus rate of gene-content change in the accessory genome. Log–log linear relationship between amino-acid differences scored in the core genome shared by all strains and gene-content differences (the number of orthologs that are either present in one genome and absent in the paired genome or present in a different copy number in the paired genome) based on reciprocal blast searches for all genomes (dashed line). Eleven independent genome pairs were used. The solid line indicates an equal rate of change.

Clades I and V are clearly distinct on the basis of a phylogenetic network based on the presence/absence of single-copy orthologs among genomes (Supplementary Figure 2). Our data set of very closely related genomes allows us to address the rate of gene-content (accessory genome) evolution relative to changes in homologous (core) genes by amino-acid substitution. The majority of lateral gene transfers are likely to be deleterious and quickly removed by purifying selection (Baltrus, 2013). Using closely related isolates ensures that little evolutionary time has passed for selection to remove deleterious or neutral changes and so is crucial for reliably estimating gene-content change (Vos *et al.*, 2015). To quantify the relative contributions of amino-acid substitutions in the core genome versus gene-content changes in the accessory genome to overall divergence, both metrics were plotted for statistically independent pairs of strains (Figure 3). The intercept indicates a rate of gene-content turnover that is an order of magnitude higher than that of amino-acid change. Although previous findings in other species have indicated that levels of gene gain and loss can be high (Hao and Golding, 2006; Nowell *et al.*, 2014), our estimate is significantly higher, which must at least in part be due to analysing more closely related genomes, but could also in part be due to *M. xanthus* having an especially fluid genome. Not detecting the presence of a gene in a genome could either be due to genuine absence or insufficient sequencing effort, but our high coverage level suggests that the high turnover in gene content is genuine. The ratio of gene-content differences to amino-acid differences decreases as a function of genetic distance between strains (Figure 3), suggesting stronger purifying selection against changes in gene content relative to protein changes in core genes. This scenario fits the observation that most changes in gene content are short-lived (Touchon *et al.*, 2009) and is analogous to the increased removal of nonsynonymous mutations relative to synonymous mutations over evolutionary time (Rocha *et al.*, 2005).

### Functional variation in accessory gene content

Differences in gene content can point towards functional and possibly ecological differentiation between strains. However, such analyses are greatly hampered by a general lack of knowledge about gene function. Approximately one-third of core genes are annotated as ‘hypothetical proteins', whereas this fraction increases to roughly two-thirds for accessory genes (*χ*^2^ Yates correction, *P*<0.0001). Differences in allelic content, most obvious when clustered in ‘islands', could also underlie ecological divergence (Shapiro *et al.*, 2012). However, no obvious clustering of SNPs could be detected in this data set (Supplementary Figure 3). A total of 245 single-copy orthologs are shared by all clade I members but not found in any member of clade V, whereas for 281 other genes the reverse is true; information on functional significance is available for only a handful of these genes (Supplementary Data).

The main consistent differences in gene content between the two clades are associated with CRISPR-Cas (clustered regularly interspaced short palindromic repeats-CRISPR-associated proteins) systems. These provide an adaptive immunity mechanism that can protect prokaryotes from invading mobile genetic elements (MGEs; Horvath and Barrangou, 2010; Westra *et al.*, 2012). MGE sequence fragments (spacers) are incorporated in a CRISPR locus, separated by repeat sequences; Cas complexes inactivate newly invading MGEs that share identity with spacers. However, it has become clear in recent years that CRISPR-Cas systems not only provide defence against MGEs but also can affect a wide variety of other cellular processes. *M. xanthus* DK1622 contains three CRISPR-Cas loci, two of type I-C and one of type III-B (Grissa *et al.*, 2007; Makarova *et al.*, 2011). One I-C locus, the *dev* operon, has a crucial role in fruiting body development in the reference strain DK1622 (Viswanathan *et al.*, 2007; Wallace *et al.*, 2014; Westra *et al.*, 2014; Rajagopalan *et al.*, 2015). All strains analysed here carry one 1-C locus but both clade I and V lack the majority of genes in the *dev* locus and the III-B locus is absent in clade I strains (Supplementary Table 4). In addition, there is extensive variation among strains in the number of CRISPR arrays, repeat length and spacer number (detailed in the Supplementary Results and Supplementary Table 4).

### Genetic diversity in a 150-kb region correlates with swarming incompatibility type

We sought to identify genetic loci potentially responsible for swarming incompatibilities by searching for patterns of genetic variation correlating with the CT patterns across strains. Because genomic regions of high SNP density may be more likely to harbour polymorphism patterns that correlate with CT groups than low-density regions, we mapped the trimmed Illumina reads derived from all 22 genomes to the reference genome of *M. xanthus* DK1622 (Supplementary Table 3) and performed sliding window genetic diversity scans (Supplementary Data File 1) for all 22 clones combined and for clade I and clade V strains separately (Supplementary Figure 3). Four regions with high SNP density were identified but only SNPs in the region approximately spanning positions 2 100 000 and 2 250 000 (that is, 150 000 or 150 kb) in reference strain *M. xanthus* DK1622 were found to cluster according to CT groupings (Supplementary Figure 4).

**Figure 4:**
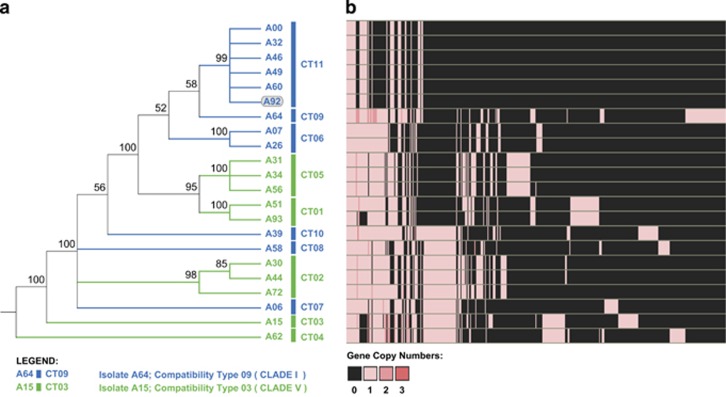
Phylogenetic analysis based on the orthologous gene-content patterns in a variable genomic region. (**a**) A maximum parsimony phylogeny based on gene-content variation in a 150 000-bp variable region that houses extensive genomic rearrangements and SNP variation. Clusters match compatibility-type (CT) groupings derived from social swarm merger assays. Bootstrap values from 100 pseudo-replication steps are shown above the branches in the tree. Branches with low support (values below 50) were collapsed. (**b**) A visual representation of both the absence (black) and presence of single (light red) and multicopy (dark red) genes in the focal 150-kb region. Genes were grouped by gene orthology classification. Strain A92 is shown in grey to highlight the incongruence of its phylogenetic position and CT grouping.

Further analysis of this region revealed an extremely high degree of gene-content variation across all strains (Supplementary Figures 5 and 6), which was found to also strongly correlate with CT groupings, as illustrated by a phylogeny based on the presence and absence patterns of all genes contained in this region across all strains (Figure 4a). Strain A92 most clearly illustrates the association between this highly variable region and CT: it groups with CT 11 strains (Figure 4; Supplementary Table 5), even though it does not cluster with these strains in the core genome-based phylogeny (Figure 1b), or it is connected with them in terms of homologous recombination (Figure 2).

The 150-kb variable region houses two of the three known complete prophages in reference strain *M. xanthus* DK1622, one of which (prophage 1) is also present in the majority of our natural isolates, except in members of the CT 11 group (Table 1). As a consequence, there is a highly significant overrepresentation of phage-related genes in this region (12 within a 107-gene region in DK1622 compared with eight in the remainder of the genome, Fisher's exact test, *P*<0.0001). Phage gene content is highly flexible and shows various degrees of gene loss (in 15 of 22 strains) but also many gene duplication events: in 9 out of 22 strains between one and four genes were duplicated at least once.

The focal region contains site-specific recombinases, transposases and rhs (rearrangement hotspot) loci, and sets of contiguous genes in this region occur across different CTs in different combinations (Supplementary Table 4). In addition, some CTs (CT2 and CT7) are present among different FineStructure populations (but not between the clades), consistent with recombination events. We identified several candidate membrane-spanning peptides present in 9 out of the 11 CTs (Supplementary Table 5). Strains A30, A58, A64, A72 and DK1622 contain an annexin repeat-containing protein (MXAN_1899) with good homology to a *Burkholderia* contact-dependent growth inhibition CDI protein (*cdiA*) implicated in between-genotype antagonism in this species (Nikolakakis *et al.*, 2012; pers com C. Hayes). The *rhs* genes found in some genomes share significant identity with *cdiA* and have also been implicated in growth inhibition in a variety of systems (Poole *et al.*, 2011; Koskiniemi *et al.*, 2013). A haem-binding protein present in some strains (MXAN_1853) could potentially also be involved, as cdiA proteins are often annotated as haemolysins, adhesins or filamentous haemagglutinins (Nikolakakis *et al.*, 2012). A variety of other genes implicated in toxin-mediated interactions is also present in this region, including a type VII secretion-associated serine protease mycosin in the three CT 11 strains and a *Clostridium* epsilon toxin EX/*Bacillus* mosquitocidal toxin in A58.

## Discussion

Our data indicate that two closely related *M. xanthus* clades inhabiting the same centimetre-scale patch of soil display strong sexual isolation, with homologous recombination occurring frequently between members within each clade, but with almost no detectable levels of genetic exchange occurring across clades. This finding contrasts with a recent study on a large set of continental and global populations of *Vibrio parahaemolyticus* also utilising FineStructure (Cui *et al.*, 2015) that did not reveal any similar barriers to homologous recombination. Several types of recombination barriers can be distinguished. First, isolation by distance will prevent strain interactions that promote recombination (Linz *et al.*, 2007). However, this scenario does not apply to the strains examined here because they co-exist at the centimetre scale. Second, sequence divergence lowers the efficiency of homologous recombination (Ansari and Didelot, 2014), but the fact that the two clades under study are very closely related speaks against this hypothesis. Third, the presence of different genetic elements, such as restriction–modification systems or CRISPR-Cas systems, could prevent successful recombination between the lineages (Budroni *et al.*, 2011). Fourth, ecological structuring, where each of the two clades inhabits a spatially and/or temporally distinct niche within the same habitat, could preclude close contact favoring genetic exchange (Vos, 2011; Shapiro and Polz, 2014)

Our population-genomic approach did not allow us to distinguish between the latter two hypotheses, especially as the mechanisms by which recombination in *M. xanthus* occurs are largely unknown. Natural transformation has been achieved under artificial conditions (Wang *et al.*, 2011) and *M. xanthus-*specific transducing phages have been isolated (Martin *et al.*, 1978) but no conjugation-mediated recombination has been found. However, it is unclear what mechanisms primarily drive recombination in nature. Mechanistic barriers to recombination, such as the differential presence of CRISPR-Cas systems that have the potential to alter the patterns of phage infection and transduction, could potentially explain the observed patterns in homologous recombination and gene content. Ecological barriers to recombination have been inferred for both a thermophilic archaeon (Cadillo-Quiroz *et al.*, 2012) and a marine proteobacterium (Shapiro *et al.*, 2012). The fact that decreased homologous recombination between the clades is associated with the increased levels of gene-content divergence could be consistent with a scenario in which the two major clades have begun to ecologically differentiate from each other. Inhabiting different spatiotemporal microniches is expected to result in the two clades being exposed to different community members from which novel genes could be recruited (Smillie *et al.*, 2011). Alternatively or in addition, the same sets of genes could be taken up by both the clades, with divergent selection resulting in subsequent differential loss.

We demonstrated that *M. xanthus* has a highly flexible genome, with changes in the accessory genome occurring at a higher rate than amino acid substitution (through mutation or homologous recombination) in the core genome (Figure 3). As our analysis featured very closely related genomes only, a recently proposed measure of genome fluidity is low for this data set compared with other species (*φ*=4%) (Kislyuk *et al.*, 2011). However, when assessing pan-genome size, that is, the total number of genes found in at least one genome, gene number did not reach a plateau, suggesting that sequencing additional genomes would uncover new genes in this population (Supplementary Figure 7) and it is expected that the global *M. xanthus* pan-genome is considerably larger.

Evidence for ecological divergence of the two clades was sought using a ‘reverse ecology' approach (Shapiro and Polz, 2014) by screening for consistent differences in accessory gene content that might reflect the differential adaptation to distinct ecological niches. However, interpretation of the potential functional significance of such gene-content differences is hindered by the high percentage of genes of unknown function. CRISPR-Cas loci were found to be among the fastest evolving parts of the genome, with important implications for their roles in protection against (or arms races with) MGEs, fruiting body development and possible interactions with prey DNA.

*M. xanthus* has diversified into a large number of mutually incompatible types, swarms of which fail to freely merge upon encounter and which are usually antagonistic when forced to undergo multicellular development in mixed groups (Fiegna and Velicer, 2005; Vos and Velicer, 2009). In a recent paper, it was shown that fruiting bodies that had formed adjacent to the ‘incompatibility zone' between colonies of two very closely related *M. xanthus* strains (belonging to a different clade within the same centimetre-scale population described here) displayed very low levels of chimerism (Rendueles *et al.*, 2015). This finding supports the hypothesis that kin discrimination could serve to prevent invasion of clonal swarms by neighbouring distinct genotypes, preventing possible social exploitation (Vos and Velicer, 2009). In the same paper, it was also shown that short-term evolution under lab conditions could generate similar kin-discrimination phenotypes. These lab-evolved kin discrimation phenotypes were found to be associated with a variety of SNPs and so must be governed by diverse genetic mechanisms (Rendueles *et al.*, 2015). Kin-discrimination patterns among the natural isolates described here however exhibit a one-to-one correlation with gene-content differences found in a large genomic region, suggesting that some of these type-specific content differences may cause kin-discrimination phenotypes in natural populations.

The eukaryotic model organisms *Dictyostelium* (Bloomfield *et al.*, 2010) and *Aspergillum* (Rydholm *et al.*, 2007) show variation in gene content (rather than allelic variation) in compatibility systems, and such variation might potentially also generate social incompatibilities in bacteria. It is unclear by what mechanisms gene-content variation in our candidate region might cause swarming incompatibilities. Recently, based on engineered laboratory strains, a lethal *M. xanthus* toxin-antitoxin system was mapped to the same variable region documented here (Dey *et al.*, 2016). The toxin is delivered across cells by outer membrane exchange (OME) mediated by the cell-surface protein TraA. Because OME appears to occur only between cells sharing similar *traA* alleles (Pathak *et al.*, 2012), this toxin could only kill competitors similar at *traA* but lacking the respective immunity gene. Strains identical at *traA* can be highly antagonistic toward one another (Pathak *et al.*, 2012; Rendueles *et al.*, 2015). It is thus possible that other OME/TraA toxins are encoded in the highly polymorphic region highlighted here that only antagonize across strains sharing the same TraA allotype.

*M. xanthus* is known to secrete a wide range of molecules into the environment (Konovalova *et al.*, 2010; Evans *et al.*, 2012; Berleman *et al.*, 2014). A thioredoxin gene is shared by all strains next to this region (Supplementary Table 5). Thioredoxins have been shown to have a role in type VI secretion systems (T6SS), which in turn are known to mediate inference competition in bacteria (Alteri *et al.*, 2013). Proteins secreted by T6SS are often part of the T6SS operon itself, but can also be encoded for elsewhere in the genome (Kapitein and Mogk, 2013). However, detailed functional studies will be necessary to elucidate the significance of genes in this region for kin discrimination.

Whole-genome analysis of very closely related natural isolates can shed light on important questions regarding the evolution of myxobacterial species that cannot be readily addressed by interspecific comparisons or experimental evolution studies. Our results will provide a basis for future work on genomic, ecological and social divergence in this fascinating model system.

## Figures and Tables

**Table 1:** Classification of natural isolates according to clade, population and compatibility type, as well as prophage sequence conservation detected in a 150-kbp variable region correlating with CT groupings

*Strain*	*Clade*	*Population*	*CT*	***Prophage 1 (reference genome's locus tags)***											
				*MXAN_1862*	*MXAN_1863*	*MXAN_1864*	*MXAN_1865*	*MXAN_1866*	*MXAN_1867*	*MXAN_1868*	*MXAN_1869*	*MXAN_1870*	*MXAN_1871*	*MXAN_1872*	*MXAN_1873*
DK1622	REF	REF	REF	1	1	1	1	1	1	1	1	1	1	1	1
A00	1	7	11												
A32	1	7	11												
A46	1	7	11												
A49	1	7	11												
A60	1	7	11												
A92	1	6	11												
A39	1	6	10	1	1	1	1	2	1	2	3	3	1	1	1
A64	1	6	9											1	2
A58	1	6	8	1	1	1	1	1	1	2	2	1			1
A06	1	6	7	2	1	1	1	1	1	2		1	1		1
A07	1	5	6										1	1	1
A26	1	5	6										1	1	1
A31	5	4	5												1
A34	5	4	5												1
A56	5	4	5												1
A62	5	3	4	1	1	1	1	1	1	2	2	1		1	1
A15	5	3	3	1	1	1	1	1	1	2	1	1	1	1	1
A72	5	3	2	1	1	1	1	1	1	2	1	1		1	1
A30	5	2	2	1	1	1	1	1	1	2	1	1		1	1
A44	5	2	2	1	1	1	1	1	1	2	1	1		1	1
A51	5	1	1												1
A93	5	1	1												1

Abbreviations: CT, compatibility type; REF, reference type; 1, 2 and 3, copy number of sequence homologues in natural isolates.
