# Supplementary material for: A barrier to homologous recombination between sympatric strains of the cooperative soil bacterium Myxococcus xanthus
Source: ISME J. 2016 Apr 5;10(10):2468–77. doi: 10.1038/ismej.2016.34 (PMC5030687; doi:10.1038/ismej.2016.34)
Supplement: Supplementary Information [file ismej201634x1.doc]

**Supplementary Information**

Wielgoss et al. “A barrier to homologous recombination between sympatric strains of the cooperative soil bacterium *Myxococcus xanthus*”

**Supplementary Methods**

Illumina-generated raw reads

The genomes of 22 representatives of two closely related *M. xanthus* clades occurring interspersed in a 16x16 cm soil population were sequenced using Illumina HiSeq technology. The raw data was purged of low quality and adapter-derived sequence in two successive steps. In the first round, reads were filtered if they contained more than 10 unknown bases (‘N’s), or more than 40 bases with phred quality lower than 20 using BGI’s in-house quality control pipeline. In the second round, all adapter sequences, read duplicates and low quality reads were removed with Sickle version 1.200 (Joshi and Fass 2011)(https://github.com/najoshi/sickle), using a minimum window quality score of 20 and discarding reads shorter than 10 bp after trimming, and Cutadapt(Martin 2011), with the -O 3 option (3' end of any reads which match the adapter sequence for 3 bp or more were trimmed. Hence, a total of >10,000,000 paired high quality 100bp reads were available for each genome, yielding a net >100-fold coverage per base pair (Supplementary Table S1).

De-novo assembly and annotation

Paired-end Illumina reads were assembled using SPAdes 2.5.0, with the “--careful” option (Bankevich et al 2012). SPAdes was run once using the recommended kmer values (-k 21,33,55,77), and again using an additional longer kmer (-k 21,33,55,77,87). The scaffolded contigs file (scaffolds.fasta) was used for all subsequent analyses. Assemblies containing likely mis-assembly errors (assessed as detailed below by comparisons with the DK 1622 genome and by mapping the reads back to the assembly) were excluded, and if both runs produced good assemblies, the one with the larger N50 value was selected for further analysis.

Scaffolds were compared to the complete genome sequence of *M. xanthus* DK1622, using *nucmer* included in MUMmer version 3.23, with the following parameters “--noextend -b=1 -c=20 -g=0” (Delcher et al 2002, Kurtz et al 2004). Scaffolds were reordered and reoriented relative to the reference genome using a custom Perl script and concatenated using a spacer sequence with both start and stop codons in all six reading frames (Tettelin et al 2005)to assist in the prediction of genes overlapping contig boundaries. The concatenated scaffolds were annotated using Prokka version 1.5.2 (Seemann 2014), which relies on both HMMER3(Finn et al 2011) and RNAmmer(Lagesen et al 2007). Importantly, by applying the “--usegenus” option, a major strength of this annotation approach comprised the generation of *Myxococcus*-specific BLAST database which included all available fully annotated complete genomes of the three species *M. xanthus* DK1622(Goldman et al 2006), *M. fulvus* FW-1(Li et al 2011), and *M. stipitatus*DSM 14675 (Huntley et al 2011). On average, 7367 coding sequences (CDSs), 76 tRNA as well as 3 different rRNA coding genes (5S, 16S, 23S) were predicted in each genome, which corresponds with expectations based on the reference genomes (Table S2). For example, the closed reference genome of strain *M. xanthus* DK1622 encodes 7331CDSs, 65 tRNAs and 3 different rRNA genes in one contig of 9.1 Mb length (4.5x coverage; Goldman et al 2006). The annotated contigs were separated and the spacer sequences removed. Assembly and annotation statistics are summarized in Supplementary Table S2 and Supplementary Figure S1. On average, individual genomes could be reliably assembled into ~80 contiguous sequences (> 500bp) with total lengths of ~9.1 Mb. The largest contigs averaged 1.1 Mb, and the assembly yielded an N50 of 617,000 bp.

Read alignment and variant detection relative to the reference genome

Reads were aligned to the reference sequence *M. xanthus* DK1622 (9.14 Mb, 68.9% GC, GenBank accession number CP000113). Read pairs were mapped to the reference sequences using Bowtie2 version 2.1.0 with the default “sensitive end-to-end” parameters “-D 15 -R 2 -N 0 -L 22 -i S,1,1.15” (Langmead and Salzberg 2012). Read pairs were tagged as “properly paired” if the distance between the 5’ ends of the two reads after alignment was between 0 and 500 bp and the relative mapping orientation was forward/reverse. To retain uniquely aligned reads only, alignments were filtered to remove reads with a mapping quality lower than 10. Read duplicates (arising from PCR amplification) were filtered to retain only a single representative using the Picard “MarkDuplicates” tool, version 1.85 (http://picard.sourceforge.net) to prevent bias in variant calling. Variant detection was performed using the GATKversion 2.1.13 “UnifiedGenotyper” package (DePristo et al 2011, McKenna et al 2010), by locally re-aligning mapped reads to avoid misalignment caused by small indels, and SNPs and small indels were identified separately. The same package was used to filter out variants with a depth of coverage less than 10 reads, or variant calls with low confidence. Variants passing this filter were annotated using snpEff version 3.2a (Cingolani 2012). IGV(Thorvaldsdóttir et al 2012) was used to visualize read coverage.

CRISPR-Cas analyses

CRISPR-CAS loci were identified using CRISPRFinder(Grissa et al 2007). CRISPRTarget(Biswas et al 2013) was used for spacer analysis and spacer BLAST was run with default options “Gap open: -10, Extend: -2, Nucleotide match: 1, Mismatch: -1,E-value: 1, Word size: 7”.

**Supplementary Results**

Functional variation in accessory genes (non-CRISPR-Cas-related)

All Clade I strains carry a glutathione S-transferase, which is implicated in a variety of functions, including defence against oxidative stress (Vuilleumier 1997). All Clade V strains carry genes involved in the biosynthesis of the toxin violacein(August et al 2000) (which is also present in *M. stipitatus*) as well as a chondroitinase-B precursor involved in the degradation of glycosaminoglycans(Huang et al 1999). All Clade I strains carry an extra copy of asparaginyl-tRNA and prolyl-tRNAsynthetases relative to Clade V, whereas a subset of Clade V strains carry an extra alanyl-tRNAsynthetase copy. Clade V carries an extra copy of a pyridoxamine 5'-phosphate oxidase, a kynurenine 3-monooxygenase, an amine oxidase and a glucosyl hydrolase.

Variation in CRISPR-Cas loci

Many of the consistent differences in gene content between the two clades are associated with CRISPR-Cas (Clustered Regularly Interspaced Short Palindromic Repeats-CRISPR Associated Proteins) systems. All strains analysed here carry one 1-C locus (here termed ‘I-C^a^’) with the III-B locus is absent in Clade I strains and both Clade I and V lack the majority of genes in the *dev* locus (here termed ‘I-C^b^’) (‘Cas Types’ in Supplementary Table S4). The recently characterized promotor region that regulates the novel inhibitor of development *devI* (MXAN_7266) in DK1622 is entirely absent from all 22 strains examined here, a general pattern observed in many other natural isolates of *M. xanthus*(Rajagopalan et al 2015). Thus, despite their proven sporulation-proficiency(Vos and Velicer 2006), the *dev*-operon is largely incomplete in our 22 focal strains, including for sequence segments required for development by DK1622 (Rajagopalan et al 2015, Viswanathan et al 2007, Wallace et al 2014, Westra et al 2014).

Intra-specific variation in the presence/absence of CRISPR repeat-spacer arrays has been documented in a variety of model systems (Andersson and Banfield 2008, Held et al 2010, Horvath et al 2008, Kuno et al 2014). Both the CRISPR array and spacer number diversity in the 22 *M. xanthus* strains conformed to this pattern of rapid evolution (Supplementary Table S4).

Clade I strains contain a very short fragment of the I-Cb Cas6 family protein MXAN_7265 (~11% of DK1622 MXAN_7265). Clade V strains contain a slightly longer (17%) fragment of the same gene, as well as short fragment (41%) of the I-Cb Cas2 family protein and a nearly complete (97%) Cas4/Cas1 fusion protein. Clade I strains contain a CRISPR array that is physically (and presumably functionally) linked to the I-Ca Cas genes. The consensus direct repeat in this array differs by one nucleotide from *M. xanthus* DK1622. In contrast to DK1622, a second CRISPR array is present with direct repeats with 100% identity to that of a *M. fulvus* HW-1 repeat but 32 bp instead of 37 bp in length, which is, as is the case in *M. fulvus*, not linked to a Cas locus. Strain A49 has an additional CRISPR array without homology to other direct repeats. In addition to the I-C^a^ arrays, two I-C^b^ arrays, one III-B CRISPR array and the *M. fulvus*-like array are also present in Clade I strains (Supplementary Table S4). Clade V strains contain two CRISPR arrays sharing an unknown direct repeat sequence (Supplementary Table S4).

Rapid spacer addition as well as extensive variation in spacer number in individual CRISPR arrays among coexisting strains has been observed in different species (Held et al 2010, Pride et al 2011, Tyson and Banfield 2008). Differences in array number, repeat length and spacer number can be observed within the Cas types described here too, allowing for further distinction between strains (CRISPR genotypes in Supplementary Table S4). Differences in CRISPR-Cas content could potentially lead to divergence in gene content via self-targeting (Dy et al 2013). However, although several instances of spacers matching self-DNA were observed, proto-spacer DNA was not found to be flanked by Protospacer Adjoining Motifs (PAMs) required for self-targeting.The identity of the spacers was investigated by collating the top hits of a BLAST screen (Biswas et al 2013). Of the combined 195 spacers from the A15 and A92 I-C^a^ CRISPR arrays (Supplementary Table S5), only the first three in each array were shared, demonstrating a rapid divergence in spacer identity. A BLAST search of the 192 unique spacers produced 70 hits (36%). Of these, 12.9% were produced by the *M. xanthus*prophage Mx8 (Orndorff et al 1983), 62.9% by bacterial chromosomes (12.9% *Myxoccocales*spp), 12.9% by eukaryotic microbes and 11.1% by eukaryotic macrobes. Because spacers are short and databases incomplete and biased, these findings are not straightforward to interpret. The majority of hits can be attributed to prokaryotes and not to MGEs (although wrongly annotated prophages and conjugative plasmids could potentially be present). DNA of a variety of soil bacteria (e.g. *Pseudomonas resinovorans* and *Streptomyces flavogriseus*) is present among the prokaryote hits, raising the hypothesis that DNA of prey bacteria might be incorporated into the CRISPR arrays of predatory myxobacteria.

Gene content in the 150kbp-variable region correlating with swarming incompatibility type

Genes present in the candidate region found to be consistently different between different Compatibility Types were tabulated for all strains and the reference DK1622 (Supplementary Table S5). In DK1622, this region is located between genes MXAN_1785 and MXAN_1908 and is present at approximately the same genomic position in all of the Tübingen genomes, with two exceptions: the first part of this region is not present at all in strains A15 and A62. Across the 19 genomes among which this region is syntenic, its gene content varies greatly, with some variation present within compatibility types (Figure 4 and Supplementary Table S5). The region is shortest in Compatibility Type 1, where it is comprised of only 40 genes (A0, A32, A46, A49, A60 and A92), and is longest in Compatibility Type 7, where it is comprised of 166 genes (A15) (Supplementary Table S5).

**Supplementary File Descriptions: Figures**

**Supplementary Fig. S1.** Summary plots of genome assembly and annotation for coding sequence (CDS) features. CDS feature numbers are plotted over, **A**, assembly sizes; **B**, number of contigs; **C**, N50; and correlation coefficients were inferred, respectively, assuming a linear model. Statistical tests were performed to assess if the correlation of the two parameters is significantly different from zero. [File type: *.docx]

**Supplementary Fig. S2**. Gene Content Neighbour-Joining Tree based on 1545 single copy orthologs presence/absence patterns for all 22 A strain genomes (SplitstreeGeneContentDistance option). [File type: *.docx]

**Supplementary Fig. S3.** Sliding window plots of % variant sites per 20,000 bp (moving in steps of 10,000 bp). **A**, all 22 genomes; **B**, the 12 Group I genomes; **C**, the 10 Group V genomes. The red coloured bar indicates the SNP spike corresponding to the 150kbp-variable region, the blue coloured bars indicate SNP spikes that are not associated with consistent differences in gene content. [File type: *.docx]

**Supplementary Fig. S4.** Screenshots from the package Integrative Genomics Viewer, displaying read coverage from each sample from Clade I and Clade V, across a 225kbp region of the *M. xanthus* DK1622 reference genome. Shown is a genomic region between positions 2.02-2.27Mbp, which includes the 150kbp-variable region spanning positions 2.10-2.25Mbp. The coloured histograms in the central section of each figure indicate the number of reads from each sample mapped to each position across the region. Samples are grouped according to compatibility type (as indicated). The top section of each screenshot indicates the position of the displayed region within the reference genome, and the lower section shows the annotated genes within the displayed region.[File type: *.docx]

**Supplementary Fig. S5.** Multiple alignment of the whole genomes of 13 A strains representative of the different compatibility type (CT) groupings and the reference strain *M. xanthus* DK1622. Red arrows depict a hotspot for genomic rearrangements between position 2.10-2.25Mbp in an otherwise largely syntenic background. [File type: *.pdf]

**Supplementary Fig. S6.** Multiple alignment of the 150kbp-variable region spanning positions 2.10-2.25Mbp for all 22 A strains and reference strain *M. xanthus* DK1622. Compatibility type (CT) groupings are highlighted on the lefthand-side. Largely and fully conserved regions are highlighted by connection lines between genomes (blue and red connectors respectively). Dotted lines depict the absence of aligned gene blocks. [File type: *.pdf]

**Supplementary Fig. S7.** Core- and pan-genome size analysis. Each genome contained an average of ~6870 genes, of which ~6400 genes are ‘core’, i.e. present in all genomes from both Clades I and V. The number of core genes was significantly higher for individual clades (~6650 and ~6700 for Clades I and V respectively), suggesting that some genes are clade-specific. The three decreasing curves indicate the number of genes found in all genomes as more genomes are added. The three increasing curves indicate the number of genes found in at least one of the genomes. All 22 genomes are considered in the black curves, whereas only genomes from Clades I and V are considered in the blue and green curves, respectively. [File type: *.docx]

**Supplementary File Descriptions: Tables**

**Supplementary Table S1.**Sequence data summary. [File type: *.docx]

**Supplementary Table S2.** Summary of assembly and annotation for each of the 22 *M. xanthus* clones. [File type: *.docx]

**Supplementary Table S3.**Summary of alignment results. [File type: *.docx]

**Supplementary Table S4.**Summary of CRISPR-Cas variation. [File type: *.docx]

**Supplementary Table S5.** Genes present in the 150kbp-variable region spanning positions 2.10-2.25Mbp of the reference genome *M. xanthus* DK1622.[File type: *.xlsx]

**Supplementary Data File Description:**

**Supplementary Data File S1.**Summary spreadsheet file covering results from genetic diversity scans across genomes. [File type: *.xlsx]

**Supplemental References**

Andersson AF, Banfield JF (2008). Virus population dynamics and acquired virus resistance in natural microbial communities. *Science***320:** 1047-1050.

August P, Grossman T, Minor C, Draper M, MacNeil I, Pemberton J *et al* (2000). Sequence analysis and functional characterization of the violacein biosynthetic pathway from Chromobacterium violaceum. *J Mol Microbiol Biotechnol***2:** 513-519.

Bankevich A, Nurk S, Antipov D, Gurevich AA, Dvorkin M, Kulikov AS *et al* (2012). SPAdes: a new genome assembly algorithm and its applications to single-cell sequencing. *J Comput Biol***19:** 455-477.

Biswas A, Gagnon JN, Brouns SJ, Fineran PC, Brown CM (2013). CRISPRTarget: Bioinformatic prediction and analysis of crRNA targets. *RNA Biol***10:** 817-827.

Cingolani P (2012). snpEff: Variant effect prediction.

Delcher AL, Phillippy A, Carlton J, Salzberg SL (2002). Fast algorithms for large-scale genome alignment and comparison. *Nucleic acids research***30:** 2478-2483.

Finn RD, Clements J, Eddy SR (2011). HMMER web server: interactive sequence similarity searching. *Nucl Acids Res***:** gkr367.

Goldman B, Nierman W, Kaiser D, Slater S, Durkin A, Eisen J *et al* (2006). Evolution of sensory complexity recorded in a myxobacterial genome. *Proc Natl Acad Sci U S A***103:** 15200-15205.

Grissa I, Vergnaud G, Pourcel C (2007). CRISPRFinder: a web tool to identify clustered regularly interspaced short palindromic repeats. *Nucl Acids Res***35:** W52-W57.

Held NL, Herrera A, Cadillo-Quiroz H, Whitaker RJ (2010). CRISPR associated diversity within a population of Sulfolobus islandicus. *Plos One***5:** e12988.

Horvath P, Romero DA, Coûté-Monvoisin A-C, Richards M, Deveau H, Moineau S *et al* (2008). Diversity, activity, and evolution of CRISPR loci in Streptococcus thermophilus. *J Bacteriol***190:** 1401-1412.

Huang W, Matte A, Li Y, Kim YS, Linhardt RJ, Su H *et al* (1999). Crystal structure of chondroitinase B from Flavobacterium heparinum and its complex with a disaccharide product at 1.7 Å resolution. *J Mol Biol***294:** 1257-1269.

Huntley S, Hamann N, Wegener-Feldbrügge S, Treuner-Lange A, Kube M, Reinhardt R *et al* (2011). Comparative genomic analysis of fruiting body formation in Myxococcales. *Mol Biol Evol***28:** 1083-1097.

Joshi N, Fass J (2011). Sickle: A sliding-window, adaptive, quality-based trimming tool for FastQ files (Version 1.33)[Software].

Kuno S, Sako Y, Yoshida T (2014). Diversification of CRISPR within coexisting genotypes in a natural population of the bloom-forming cyanobacterium *Microcystis aeruginosa*. *Microbiology***160:** 903-916.

Kurtz S, Phillippy A, Delcher AL, Smoot M, Shumway M, Antonescu C *et al* (2004). Versatile and open software for comparing large genomes. *Genome Biol***5:** R12.

Lagesen K, Hallin P, Rødland EA, Stærfeldt H-H, Rognes T, Ussery DW (2007). RNAmmer: consistent and rapid annotation of ribosomal RNA genes. *Nucl Acids Res* **35:** 3100-3108.

Langmead B, Salzberg SL (2012). Fast gapped-read alignment with Bowtie 2. *Nature Meth***9:** 357-359.

Li Z-F, Li X, Liu H, Liu X, Han K, Wu Z-H *et al* (2011). Genome sequence of the halotolerant marine bacterium *Myxococcus fulvus* HW-1. *J Bacteriol***193:** 5015-5016.

Martin M (2011). Cutadapt removes adapter sequences from high-throughput sequencing reads. *EMBnet journal***17:** pp. 10-12.

Orndorff P, Stellwag E, Starich T, Dworkin M, Zissler J (1983). Genetic and physical characterization of lysogeny by bacteriophage MX8 in *Myxococcus xanthus*. *J Bacteriol***154:** 772-779.

Rajagopalan R, Wielgoss S, Lippert G, Velicer GJ, Kroos L (2015). *devI* is an Evolutionarily Young Negative Regulator of *Myxococcus xanthus* Development. *J Bacteriol***197**: 1249-1262.

Seemann T (2014). Prokka: rapid prokaryotic genome annotation. *Bioinformatics***:** btu153.

Tettelin H, Masignani V, Cieslewicz MJ, Donati C, Medini D, Ward NL *et al* (2005). Genome analysis of multiple pathogenic isolates of *Streptococcus agalactiae*: implications for the microbial “pan-genome”. *Proc Natl Acad Sci USA***102:** 13950-13955.

Thorvaldsdóttir H, Robinson JT, Mesirov JP (2012). Integrative Genomics Viewer (IGV): high-performance genomics data visualization and exploration. *Briefings in bioinformatics***:** bbs017.

Viswanathan P, Murphy K, Julien B, Garza AG, Kroos L (2007). Regulation of dev, an operon that includes genes essential for Myxococcus xanthus development and CRISPR-associated genes and repeats. *J Bacteriol* **189:** 3738-3750.

Vos M, Velicer GJ (2006). Genetic Population Structure of the Soil Bacterium Myxococcus xanthus at the Centimeter Scale. *Appl EnvMicrobiol***72:** 3615-3625.

Vuilleumier S (1997). Bacterial glutathione S-transferases: what are they good for? *Journal of bacteriology***179:** 1431.

Wallace RA, Black WP, Yang X, Yang Z (2014). A CRISPR with roles in Myxococcus xanthus development and exopolysaccharide production. *J Bacteriol***:** JB. 02035-02014.

Westra ER, Buckling A, Fineran PC (2014). CRISPR-Cas systems: beyond adaptive immunity. *Nature Rev Microbiol***12:** 317-326.
