# Supplementary figures and images for: A barrier to homologous recombination between sympatric strains of the cooperative soil bacterium Myxococcus xanthus
Source: ISME J. 2016 Apr 5;10(10):2468–77. doi: 10.1038/ismej.2016.34 (PMC5030687; doi:10.1038/ismej.2016.34)

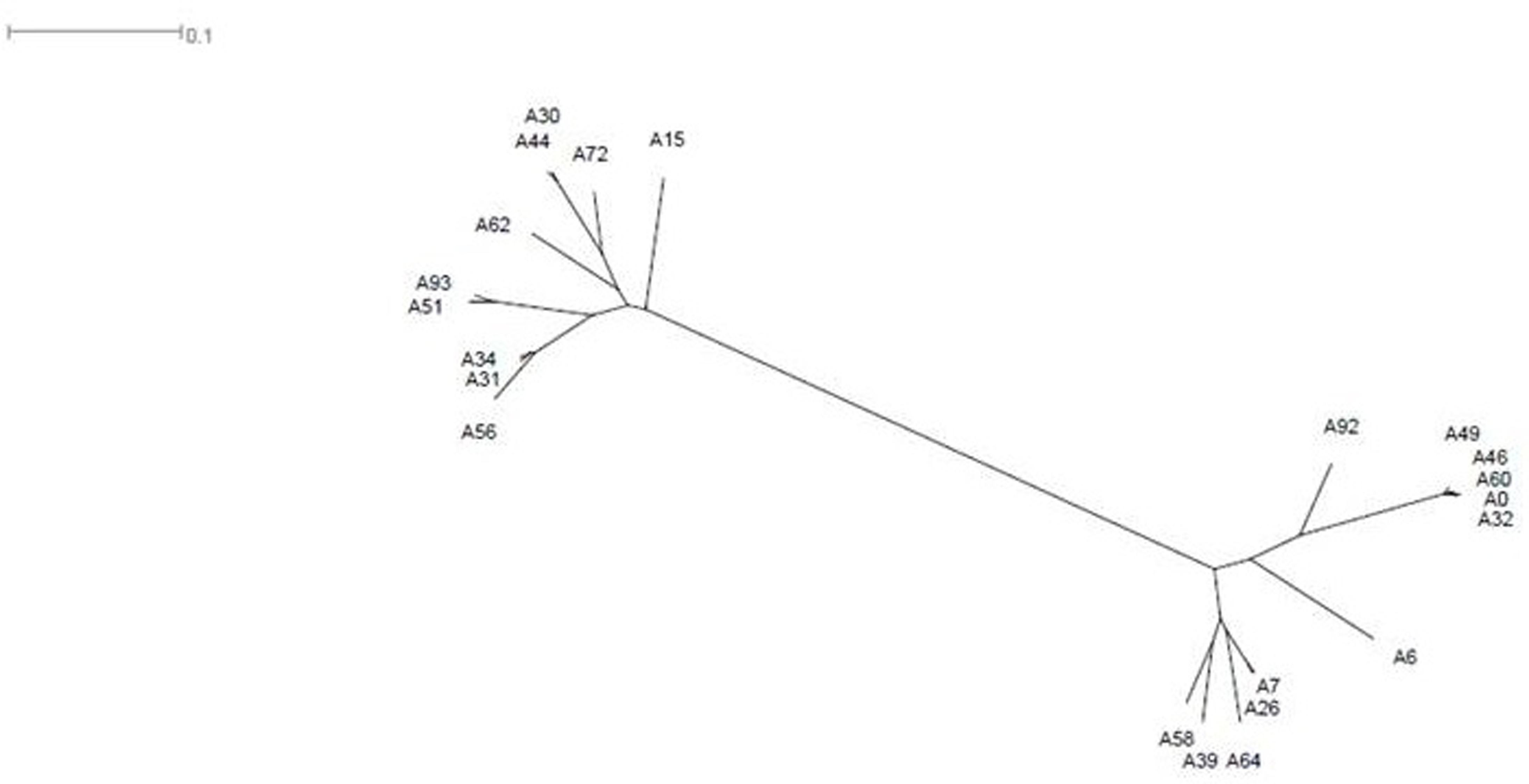

Supplement: Supplementary Figure S2 [file ismej201634x3.tif]

Supplementary Figure S4.


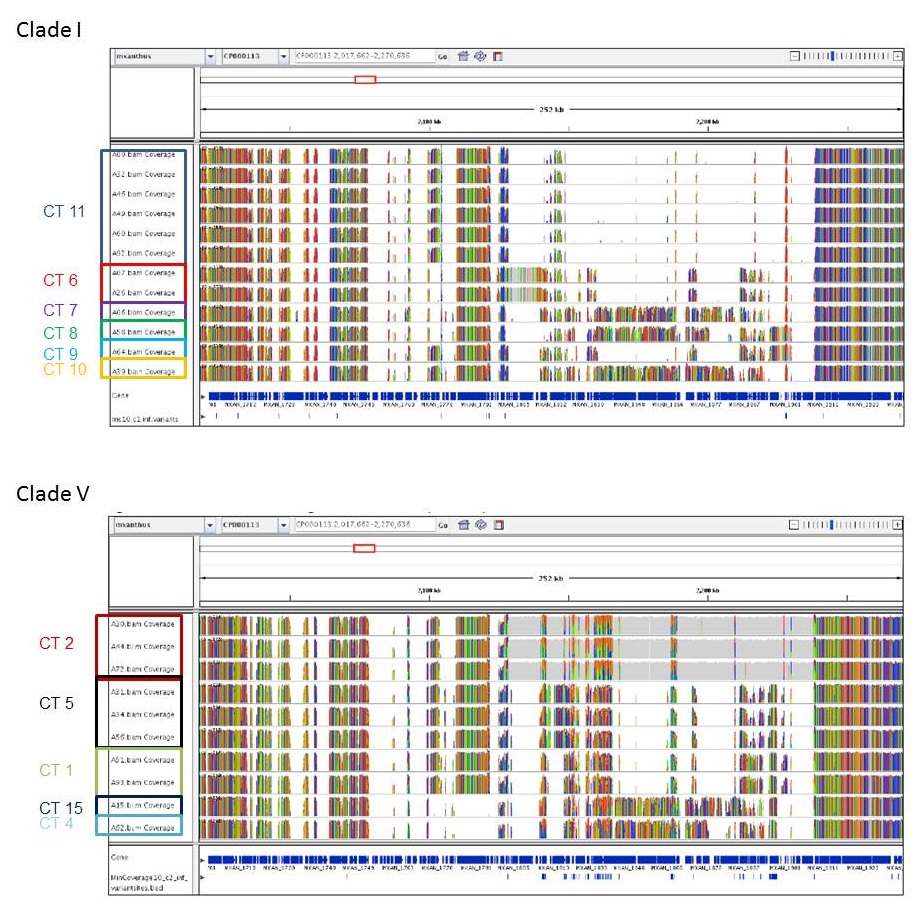

Supplement: Supplementary Figure S4 [file ismej201634x5.doc]

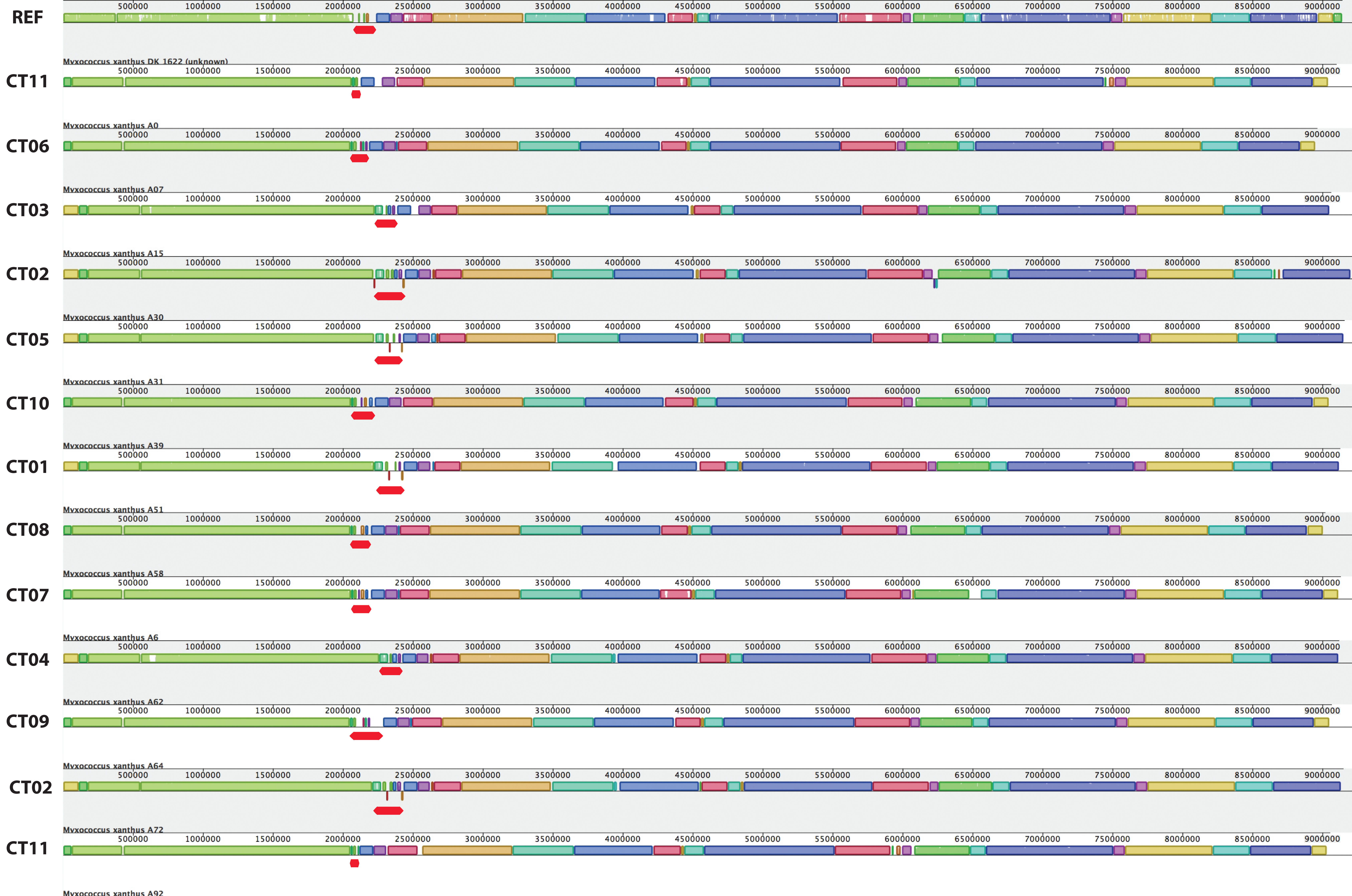

Supplement: Supplementary Figure S5 [file ismej201634x6.pdf]
