# Supplementary material for: A barrier to homologous recombination between sympatric strains of the cooperative soil bacterium Myxococcus xanthus
Source: ISME J. 2016 Apr 5;10(10):2468–77. doi: 10.1038/ismej.2016.34 (PMC5030687; doi:10.1038/ismej.2016.34)
Supplement: Supplementary Figure S7 [file ismej201634x8.doc]

Supplementary Fig. S7
