# Supplementary material for: A barrier to homologous recombination between sympatric strains of the cooperative soil bacterium Myxococcus xanthus
Source: ISME J. 2016 Apr 5;10(10):2468–77. doi: 10.1038/ismej.2016.34 (PMC5030687; doi:10.1038/ismej.2016.34)
Supplement: Supplementary Table S1 [file ismej201634x9.doc]

Supplementary Table S1. Sequence data summary.

| Sample ID | Clade | Compatibility type (CT) | Raw Data (Mb) | Adapter (%) | Duplication (%) | Total Reads | Filtered Reads (%) | Low Quality Filtered Reads (%) | Clean Data (Mb) |
| --- | --- | --- | --- | --- | --- | --- | --- | --- | --- |
| A30 | V | 10 | 1131 | 0.85 | 0.08 | 11,306,580 | 10.8 | 5.45 | 1009 |
| A44 | V | 10 | 1132 | 1.11 | 0.08 | 11,315,038 | 11.6 | 5.80 | 1000 |
| A72 | V | 10 | 1155 | 1.25 | 0.08 | 11,552,020 | 12.7 | 6.46 | 1008 |
| A31 | V | 11 | 1128 | 1.85 | 0.06 | 11,282,872 | 11.3 | 5.22 | 1001 |
| A34 | V | 11 | 1129 | 1.28 | 0.07 | 11,286,002 | 11.4 | 5.60 | 1000 |
| A56 | V | 11 | 1160 | 1.67 | 0.07 | 11,601,130 | 13.4 | 6.56 | 1005 |
| A51 | V | 9 | 1161 | 1.24 | 0.08 | 11,611,682 | 13.1 | 6.65 | 1009 |
| A93 | V | 9 | 1348 | 0.62 | 0.06 | 13,483,826 | 25.3 | 15.1 | 1007 |
| A15 | V | 7 | 1125 | 0.93 | 0.09 | 11,247,686 | 10.6 | 5.32 | 1005 |
| A62 | V | 8 | 1135 | 0.45 | 0.12 | 11,346,742 | 11.6 | 6.16 | 1003 |
| A00 | I | 1 | 1132 | 0.83 | 0.08 | 11,318,534 | 11.2 | 5.74 | 1005 |
| A32 | I | 1 | 1141 | 0.65 | 0.09 | 11,407,536 | 11.5 | 5.99 | 1010 |
| A46 | I | 1 | 1144 | 2.17 | 0.07 | 11,443,106 | 12.0 | 5.48 | 1007 |
| A49 | I | 1 | 1134 | 1.02 | 0.07 | 11,342,394 | 11.7 | 5.89 | 1002 |
| A60 | I | 1 | 1131 | 0.36 | 0.10 | 11,306,428 | 11.3 | 6.03 | 1003 |
| A92 | I | 1 | 1143 | 0.68 | 0.10 | 11,432,890 | 12.0 | 6.29 | 1006 |
| A07 | I | 3 | 1151 | 0.92 | 0.06 | 11,512,878 | 12.6 | 6.54 | 1006 |
| A26 | I | 3 | 1138 | 1.02 | 0.08 | 11,383,698 | 11.6 | 5.88 | 1006 |
| A06 | I | 2 | 1134 | 0.55 | 0.09 | 11,340,604 | 11.0 | 5.78 | 1009 |
| A58 | I | 4 | 1146 | 0.33 | 0.10 | 11,463,876 | 12.0 | 6.47 | 1009 |
| A64 | I | 5 | 1150 | 0.49 | 0.10 | 11,495,024 | 12.3 | 6.57 | 1008 |
| A39 | I | 6 | 1134 | 0.78 | 0.08 | 11,340,668 | 11.0 | 5.63 | 1009 |
