# Supplementary material for: A barrier to homologous recombination between sympatric strains of the cooperative soil bacterium Myxococcus xanthus
Source: ISME J. 2016 Apr 5;10(10):2468–77. doi: 10.1038/ismej.2016.34 (PMC5030687; doi:10.1038/ismej.2016.34)
Supplement: Supplementary Table S2 [file ismej201634x10.doc]

Supplementary Table S2. Summary of assembly and annotation for each of the 22 *M. xanthus* clones.

|  |  |  |  |  |  |  |  | Annotated features | | | |
| --- | --- | --- | --- | --- | --- | --- | --- | --- | --- | --- | --- |
| Sample ID | Clade | Compatibility type (CT) | Scaffolds | Total assembly size (bp) | Largest contig (bp) | N50 (bp) | N90 (bp) | CDS | tRNA | rRNA | tmRNA |
| A30 | V | 10 | 51 | 9,211,699 | 1,472,594 | 832,591 | 247,673 | 7,496 | 74 | 3 | 1 |
| A44 | V | 10 | 58 | 9,212,385 | 1,488,054 | 914,438 | 198,066 | 7,494 | 74 | 3 | 1 |
| A72 | V | 10 | 100 | 9,147,366 | 1,468,334 | 829,662 | 251,921 | 7,402 | 74 | 3 | 1 |
| A31 | V | 11 | 69 | 9,157,386 | 1,468,835 | 621,865 | 140,752 | 7,393 | 74 | 3 | 1 |
| A34 | V | 11 | 60 | 9,155,522 | 1,468,479 | 651,468 | 189,349 | 7,397 | 74 | 3 | 1 |
| A56 | V | 11 | 80 | 9,195,084 | 963,745 | 423,866 | 155,821 | 7,431 | 75 | 3 | 1 |
| A51 | V | 9 | 82 | 9,127,814 | 1,468,749 | 550,621 | 167,004 | 7,356 | 74 | 3 | 1 |
| A93 | V | 9 | 225 | 9,184,351 | 1,468,546 | 623,875 | 167,025 | 7,362 | 76 | 4 | 1 |
| A15 | V | 7 | 53 | 9,061,139 | 1,468,496 | 791,664 | 176,439 | 7,339 | 74 | 3 | 1 |
| A62 | V | 8 | 54 | 9,118,714 | 1,468,567 | 497,517 | 297,531 | 7,391 | 75 | 3 | 1 |
| A00 | I | 1 | 83 | 9,098,728 | 1,078,672 | 623,126 | 133,523 | 7,411 | 79 | 3 | 1 |
| A32 | I | 1 | 81 | 9,095,029 | 932,114 | 550,093 | 133,582 | 7,413 | 78 | 3 | 1 |
| A46 | I | 1 | 84 | 9,097,159 | 1,078,672 | 623,180 | 130,908 | 7,406 | 79 | 3 | 1 |
| A49 | I | 1 | 85 | 9,093,575 | 931,834 | 550,338 | 130,909 | 7,399 | 79 | 3 | 1 |
| A60 | I | 1 | 77 | 9,094,441 | 1,079,226 | 623,200 | 130,928 | 7,407 | 78 | 3 | 1 |
| A92 | I | 1 | 72 | 9,053,119 | 999,086 | 603,898 | 133,933 | 7,354 | 79 | 3 | 1 |
| A07 | I | 3 | 69 | 8,961,307 | 999,106 | 550,340 | 133,953 | 7,192 | 78 | 3 | 1 |
| A26 | I | 3 | 71 | 8,960,271 | 999,086 | 623,184 | 133,933 | 7,173 | 79 | 3 | 1 |
| A06 | I | 2 | 79 | 9,129,421 | 932,023 | 457,701 | 135,024 | 7,422 | 79 | 3 | 1 |
| A58 | I | 4 | 70 | 9,018,593 | 932,088 | 451,485 | 135,282 | 7,272 | 79 | 3 | 1 |
| A64 | I | 5 | 76 | 9,064,988 | 931,024 | 550,358 | 130,928 | 7,262 | 78 | 3 | 1 |
| A39 | I | 6 | 77 | 9,061,522 | 998,187 | 620,483 | 131,037 | 7,320 | 79 | 3 | 1 |
