# Supplementary material for: A barrier to homologous recombination between sympatric strains of the cooperative soil bacterium Myxococcus xanthus
Source: ISME J. 2016 Apr 5;10(10):2468–77. doi: 10.1038/ismej.2016.34 (PMC5030687; doi:10.1038/ismej.2016.34)
Supplement: Supplementary Table S3 [file ismej201634x11.doc]

Supplementary Table S3. Summary of the alignment results.

| Sample ID | Group | CT | Reads to align^1^ | Aligned readsbefore filtering (%)^2^ | Aligned reads after filtering (%)^3^ |
| --- | --- | --- | --- | --- | --- |
| A30 | V | 10 | 10,090,000 | 8,408,054 (83.3) | 7,563,691 (75.0) |
| A44 | V | 10 | 10,000,000 | 8,320,722 (83.2) | 7,446,265 (74.5) |
| A72 | V | 10 | 10,080,000 | 8,408,789 (83.4) | 7,516,679 (74.6) |
| A31 | V | 11 | 10,010,000 | 8,334,411 (83.3) | 7,476,586 (74.7) |
| A34 | V | 11 | 10,000,000 | 8,327,947 (83.3) | 7,450,339 (74.5) |
| A56 | V | 11 | 10,050,000 | 8,181,322 (81.4) | 7,311,909 (72.8) |
| A51 | V | 9 | 10,090,000 | 8,347,767 (82.7) | 7,416,593 (73.5) |
| A93 | V | 9 | 10,070,000 | 8,319,392 (82.6) | 7,341,511 (72.9) |
| A15 | V | 7 | 10,050,000 | 8,418,762 (83.8) | 7,558,126 (75.2) |
| A62 | V | 8 | 10,030,000 | 8,317,673 (82.9) | 7,327,798 (73.1) |
| A00 | I | 1 | 10,050,000 | 8,329,422 (82.9) | 7,476,166 (74.4) |
| A32 | I | 1 | 10,100,000 | 8,360,326 (82.8) | 7,470,138 (74.0) |
| A46 | I | 1 | 10,070,000 | 8,364,390 (83.1) | 7,485,176 (74.3) |
| A49 | I | 1 | 10,020,000 | 8,288,891 (82.7) | 7,417,394 (74.0) |
| A60 | I | 1 | 10,030,000 | 8,281,365 (82.6) | 7,377,276 (73.6) |
| A92 | I | 1 | 10,060,000 | 8,346,856 (83.0) | 7,418,088 (73.7) |
| A07 | I | 3 | 10,060,000 | 8,422,421 (83.7) | 7,509,744 (74.7) |
| A26 | I | 3 | 10,060,000 | 8,452,335 (84.0) | 7,532,544 (74.9) |
| A06 | I | 2 | 10,090,000 | 8,363,475 (82.9) | 7,435,418 (73.7) |
| A58 | I | 4 | 10,090,000 | 8,436,849 (83.6) | 7,480,226 (74.1) |
| A64 | I | 5 | 10,080,000 | 8,326,578 (82.60) | 7,409,418 (73.51) |
| A39 | I | 6 | 10,090,000 | 8,447,826 (83.72) | 7,557,806 (74.90) |

^1^ Sum of all R1 and R2 reads used in the alignment

^2^Reads that align to the reference genome, before removing low mapping quality reads and redundant duplicate reads (% of all reads used in alignment)

^3^Reads that align to the reference genome, after removing low mapping quality reads (MAPQ < 11) and redundant duplicate reads (% of all reads used in alignment)
