# Supplementary material for: A barrier to homologous recombination between sympatric strains of the cooperative soil bacterium Myxococcus xanthus
Source: ISME J. 2016 Apr 5;10(10):2468–77. doi: 10.1038/ismej.2016.34 (PMC5030687; doi:10.1038/ismej.2016.34)
Supplement: Supplementary Table S4 [file ismej201634x12.doc]

Supplementary Table S4.Summary of CRISPR-Cas variation.

| **Group** | **Cas type** | **CRISPR genotype^*^** | **genome** | **n spacers** | **repeat l.** | **direct repeat (dr) consensus** | **best dr hit (location)** |
| --- | --- | --- | --- | --- | --- | --- | --- |
| I | - I-Ca | α | A0, A32, A46, | 3 | 37 | ATCTGCTTCACGAAGTCGGGCGTGACGCCGTGGATGC | *M. fulvus* HW-1^***^ |
|  | - partial I-Cb (1/7 genes) |  | A60 | 84 | 37 | GTTTCAACCCACGCTCCTCGCGTTCACGAGGAGCGAC | DK1622(I-Ca) |
|  |  | β | A92 | 3 | 28 | ATCTGCTTCACGAACTCGGGCGTGACGC | *M. fulvus* HW-1^***^ |
|  |  |  |  | *85^**^* | 37 | GTTTCAACCCACGCTCCTCGCGTTCACGAGGAGCGAC | DK1622 (I-Ca) |
|  |  | γ | A6, A7, A26, | 3 | 28 | ATCTGCTTCACGAACTCGGGCGTGACGC | *M. fulvus* HW-1^***^ |
|  |  |  | A39, A58, A64 | 84 | 37 | GTTTCAACCCACGCTCCTCGCGTTCACGAGGAGCGAC | DK1622 (I-Ca) |
|  |  | δ | A49 | 3 | 37 | ATCTGCTTCACGAAGTCGGGCGTGACGCGGTGGATGC | *M. fulvus* HW-1^***^ |
|  |  |  |  | 84 | 37 | GTTTCAACCCACGCTCCTCGCGTTCACGAGGAGCGAC | DK1622 (I-Ca) |
|  |  |  |  | 3 | 28 | CACGAGGCTCATTCGCGCGCGGGCCGCC | no hits |
| V | - I-Ca | ε | A31, A34, A56, | 3 | 37 | ATCTGCTTCACGAAGTCGGGCGTGACGCGGTGGATGC | *M. fulvus* HW-1^***^ |
|  | - partial I-Cb (3/7 genes) |  |  | *90* | 37 | GTTTCAACCCACGCTCCTCGCGTTCACGAGGAGCGAC | DK1622 (I-Ca) |
|  | - III-B |  |  | 36 | 36 | GTGCTCAACGCCTTTCGGCATCACGGCGAGCGGGAC | DK1622 (I-Cb) |
|  |  |  |  | 30 | 36 | GTGCTCAACGCCTTTCGGCATCACGGCGAGCGGGAC |  |
|  |  |  |  | 60 | 36 | GTGCTCAACGCCTCCCGGCATCACGGCGAGCGGCAC | DK1622 (III-B) |
|  |  |  |  | 3 | 24 | CCGGGAATGACGGGCGCACCGCCT | no hits |
|  |  |  |  | 6 | 24 | CCGGGAATGACGGGCGCACCGCCT |  |
|  |  | ζ | A15, A30, A44, | 3 | 37 | ATCTGCTTCACGAAGTCGGGCGTGACGCGGTGGATGC | *M. fulvus* HW-1^***^ |
|  |  |  | A51, A72, A93 | *110^**^* | 37 | GTTTCAACCCACGCTCCTCGCGTTCACGAGGAGCGAC | DK1622 (I-Ca) |
|  |  |  |  | 36 | 36 | GTGCTCAACGCCTTTCGGCATCACGGCGAGCGGGAC | DK1622 (I-Cb) |
|  |  |  |  | 30 | 36 | GTGCTCAACGCCTTTCGGCATCACGGCGAGCGGGAC |  |
|  |  |  |  | 60 | 36 | GTGCTCAACGCCTCCCGGCATCACGGCGAGCGGCAC | DK1622 (III-B) |
|  |  |  |  | 3 | 24 | CCGGGAATGACGGGCGCACCGCCT | no hits |
|  |  |  |  | 6 | 24 | CCGGGAATGACGGGCGCACCGCCT |  |
|  |  | η | A62 | 3 | 37 | ATCTGCTTCACGAAGTCGGGCGTGACGCGGTGGATGC | *M. fulvus* HW-1^***^ |
|  |  |  |  | *112* | 37 | GTTTCAACCCACGCTCCTCGCGTTCACGAGGAGCGAC | DK1622 (I-Ca) |
|  |  |  |  | 36 | 36 | GTGCTCAACGCCTTTCGGCATCACGGCGAGCGGGAC | DK1622 (I-Cb) |
|  |  |  |  | 30 | 36 | GTGCTCAACGCCTTTCGGCATCACGGCGAGCGGGAC |  |
|  |  |  |  | 60 | 36 | GTGCTCAACGCCTCCCGGCATCACGGCGAGCGGCAC | DK1622 (III-B) |
|  |  |  |  | 3 | 24 | CCGGGAATGACGGGCGCACCGCCT | no hits |
|  |  |  |  | 6 | 24 | CCGGGAATGACGGGCGCACCGCCT |  |
| NA | I-C a | NA | DK1622 | 57 | 37 | GTTTCAACCCACGCTCCCCGCGTTCACGAGGAGCGAC | I-Ca |
|  | I-C b |  |  | 8 | 36 | GTGCTCAACGCCTTTCGGCATCACGGCGAGCGGGAC | I-Cb |
|  | III-B |  |  | 22 | 36 | GTGCTCAACGCCTTTCGGCATCACGGCGAGCGGGAC |  |
|  |  |  |  | 52 | 36 | GTGCTCAACGCCTTCCGGCATCACGGCGAGCGGCAC | III-B |

Questionable CRISPRs (structures having only two or three DRs or structures where the repeated motifs are not 100% identical) were excluded from analysis. DK1622 has been added as a reference. Cas types are defined based on the Cas genes presence/absence; CRISPR genotypes are defined based on the presence/absence and identity of consensus direct repeat sequences and spacer number. *= It is worth noting that relative to CRISPR genotype α, type β differs by the addition of a single spacer, type γ differs by the shortening of the direct repeat by 9 bp (having identical spacers) and type δ differs by the addition of an entire CRISPR array, suggesting that these different types of changes can occur at similar evolutionary time scales in *M. xanthus*. **= not linked to a Cas locus, ***= used for spacer BLAST (see main text), italicized spacer numbers indicate spacer addition relative to other CRISPR types
