# Supplementary material for: A barrier to homologous recombination between sympatric strains of the cooperative soil bacterium Myxococcus xanthus
Source: ISME J. 2016 Apr 5;10(10):2468–77. doi: 10.1038/ismej.2016.34 (PMC5030687; doi:10.1038/ismej.2016.34)
Supplement: Supplementary Figure S6 [file ismej201634x7.pdf]

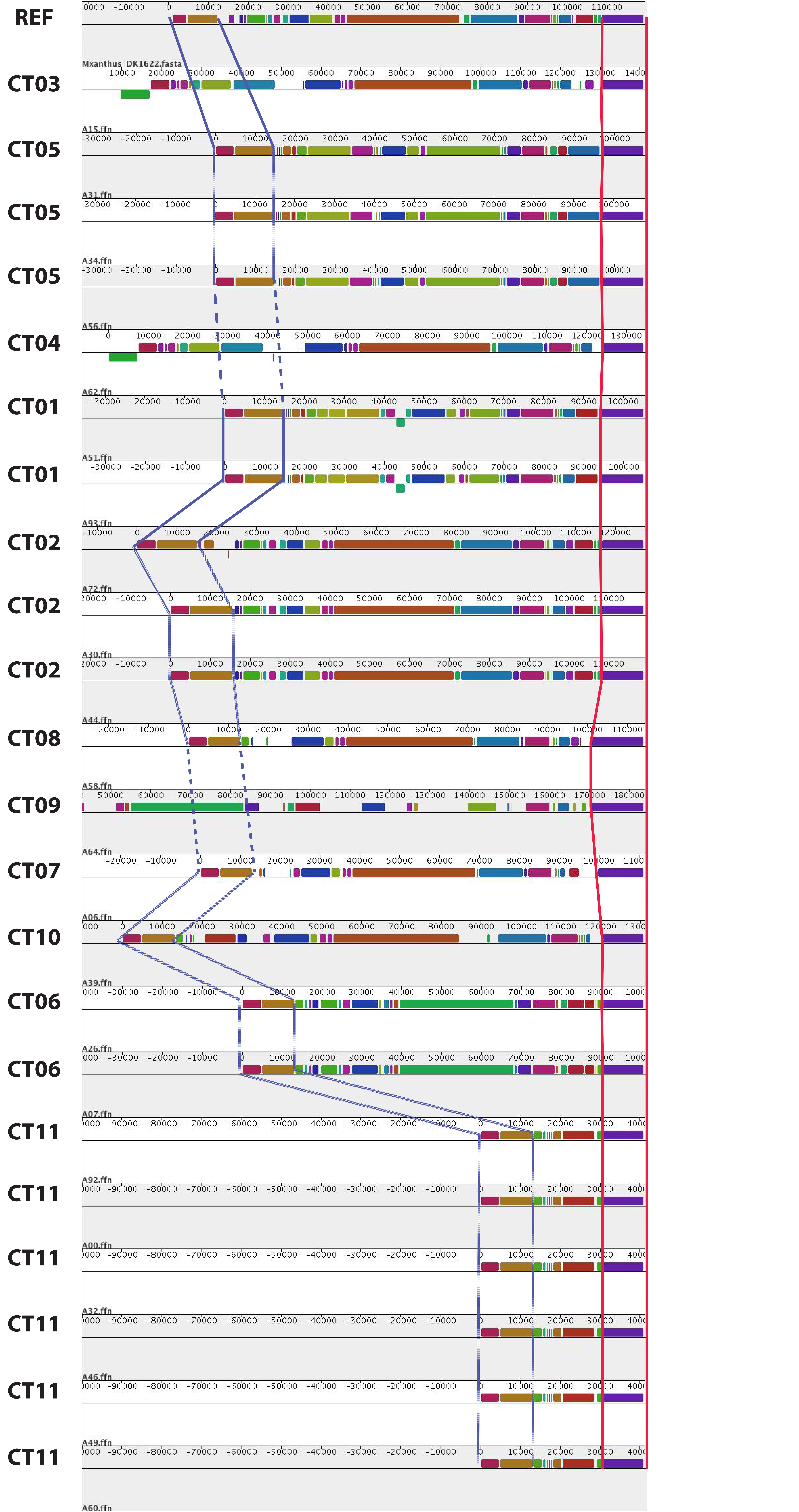
REF
Mxanthus_DK1622.fasta
CT03
A15.ffn
CT05
A31.ffn
CT05
A34.ffn
CT05
A56.ffn
CT04
A62.ffn
CT01
A51.ffn
CT01
A93.ffn
CT02
A72.ffn
CT02
A30.ffn
CT02
A44.ffn
CT08
A58.ffn
CT09
A64.ffn
CT07
A06.ffn
CT10
A39.ffn
CT06
A26.ffn
CT06
A07.ffn
CT11
A92.ffn
CT11
A00.ffn
CT11
A32.ffn
CT11
A46.ffn
CT11
A49.ffn
CT11
A60.ffn
